# Assessing bioenergetic functions from isolated mitochondria in Drosophila melanogaster

**DOI:** 10.14440/jbm.2016.112

**Published:** 2016-06-08

**Authors:** Wen C. Aw, Rijan Bajracharya, Samuel G. Towarnicki, J. William O. Ballard

**Affiliations:** School of Biotechnology and Biomolecular Science, University of New South Wales, Sydney 2052, Australia

**Keywords:** Drosophila, mitochondrial isolation, bioenergetics, Seahorse XF, complex I, larvae

## Abstract

Mitochondria are involved in generating more than 90 percent of cellular energy and are responsible for many cellular processes such as metabolism, cell signalling, apoptosis and ageing. Currently, there are a number of different experimental approaches employed to measure mitochondrial health and function. Here, we demonstrate a novel approach that quantifies substrate induced mitochondrial respiration from Drosophila. This protocol is optimized for mitochondria isolated from third instar larvae, and can also be used for mitochondria isolated from adult thoraces. This procedure outlines how to perform high throughput and high resolution mitochondria specific measurements for state II, state III, state IV_O_ respiration and residual oxygen consumption.

## BACKGROUND

The primary goal of this study is to develop a simple protocol that can be used to determine the basic bioenergetics functions of mitochondria isolated from Drosophila flies using the XF analyser (Seahorse Bioscience). Previous publications have developed protocols for cell lines [1,2] and mitochondria isolated from mouse muscle [3], mouse liver [4] and mouse heart [5]. However, no high-throughput method has been developed to measure the mitochondrial functions in Drosophila, despite its common use as a disease model and genetic tool. A secondary goal of this study is to compare the mitochondrial uncoupling agent carbonyl cyanide-4-phenylhydrazone (FCCP) with (2-fluorophenyl){6-[(2-fluorophenyl)amino](1,2,5-oxadiazolo[3,4-e]pyrazin-5-yl)}amine (BAM 15). FCCP is the most commonly used uncoupling agent, which can be cytotoxic in high concentration. In contrast, BAM15 is a new uncoupling agent that does not depolarize the plasma membrane [6].

To date, two different experimental approaches have been employed to measure mitochondrial health and function in flies. The traditional Clark-type oxygen electrode is based on oxygen dissolved in liquid or gas phase in the sample chamber and signal is detected by polarography [7]. Clark-type oxygen electrodes have provided a wealth of information but are limited in flexibility, sensitivity and throughput. A second method utilizes the Oxygraph-2k (Oroboros) [8]. The Oxygraph-2K is an advanced polarimeter that has two chambers for parallel measurements. Its advantage is the oxygen leak from the instrument is reduced and has increased detection sensitivity. However, throughput is low and routine calibration of the instrument is required [9,10].

Here, we utilize the XF24 analyzer to measure mitochondrial function in Drosophila through determination of the oxygen consumption rate (OCR). The Seahorse XF extracellular flux analyzer is based on detection of OCR with oxygen-sensing fluorophores and extracellular acidification rate (ECAR) with pH sensor simultaneously in the same population of intact cells or isolated mitochondria [11]. Seahorse XF has specific strengths when compared to Oxygraph-2k. In comparison, the Seahorse XF has higher throughput allowing for the simultaneous assessment of multiple conditions and additional replicates. Additionally, OCR and ECAR can be measured in parallel to determine the mitochondrial respiration and glycolytic activity [11,12]. There are, however, also disadvantages to the XF24. Importantly, the XF is less flexible than the Oroborus where the user can inject sequential additions of each substrate. Also, the current version of the XF can only operate at a minimum of 7°C above ambient. To overcome this limitation we placed the XF analyzer in a temperature-controlled incubator set to 15°C and conducted all assays at 23°C (though this can be modified according to the user’s needs). A previous advantage of the Oroborus was that it could assay mitochondrial functions in permeabilized fibers. However, a recent technical note from Seahorse would appear to have overcome this issue [13].

Here we outline a protocol optimized for mitochondrial extracts from *Drosophila melanogaster* larvae, which can also be used in adults. In this protocol, we assessed mitochondrial respiration by adding four injection compounds. The basal respiration (State II) is monitored before addition of ADP. Basal respiration is controlled by proton leak and substrate oxidation. The respiration of state III was assessed by injection of ADP to stimulate ATP turnover. State III respiration reflects OXPHOS activity under saturated substrate, oxygen and ADP levels. State III respiration can be terminated by adding the ATP synthase inhibitor oligomycin, to obtain a state IVo rate. In the presence of oligomycin, respiration is highly dependent on proton leak [2]. The injection of the uncoupling agents BAM15 or FCCP re-establishes proton flux, and gives rise to maximum capacity of ATP generation or oxygen consumption [6,14]. Finally, the residual oxygen consumption can be measured by injection of electron transport system (ETS) inhibitors rotenone/antimycin A. These inhibitors will stop all respiration/oxygen consumption by preventing the transfer of protons in the ETS. The mitochondrial respiratory control ratio (RCR) is calculated as state III/state II or state III/state IVo (State II is used as “pseudo state IV” measurement when oligomycin is not injected). A high RCR implies that the mitochondria have a high capacity for substrate oxidation and ATP turnover.

**Table 1.** XF instrument protocol for OCR measurement of isolated mitochondria from Drosophila.

Command	Time	Port
Calibrate Probes	-	-
Mix	0m 30s	
Measure	4m 0s	
Mix	0m 30s	
Measure	4m 0s	
Mix	0m 30s	
Inject A	-	ADP
Mix	0m 30s	
Measure	10m 0s	
Mix	0m 30s	
Inject B	-	Oligomycin
Mix	0m 30s	
Measure	4m 0s	
Mix	0m 30s	
Inject C	-	BAM 15/ FCCP
Mix	0m 30s	
Measure	4m 0s	
Mix	0m 30s	
Inject D	-	Antimycin A + Rotenone
Mix	0m 30s	
Measure	4m 0s	

## MATERIALS

### Reagents

• One-cc syringe filled with 5.0 cm^2^ of cotton
• Plastic microtube pestle (Dounce)
• Bradford reagent (Sigma B6916)
• 1 mg/ml protein standard (Sigma P0914)
• XF24 FluxPak (SeahorseBioscience, MA, USA)
**NOTES**: The XF24 FluxPak contains 18 XF24 extracellular flux assay kits. Each assay kit contains one sensor cartridge, one utility plate (for sensor cartridge calibration), and one lid. The XF24 FluxPak includes 20 polystyrene XF cell culture microplates for mitochondria seeding purpose and one bottle of 500 ml of XF24 calibrant solution (pH7.4).

### Recipes

• Isolation buffer: 154 mM KCl, 1 mM EDTA, pH 7.4
• Mitochondrial Assay Solution (MAS): 115 mM KCl (Sigma P9333), 10 mM KH_2_PO_4_ (Sigma P9791), 2 mM MgCl_2_ (Sigma M1028), 3 mM HEPES (Sigma H0887), 1mM EGTA (Sigma E4378), FA-free BSA 0.2% (A7511), adjust solution to pH 7.2 using 5 N KOH.
CAUTIONS: The presence of BSA in the MAS solution preserves mitochondrial coupling. It is essential to have a concentration of 0.2% BSA in the MAS solution.
• Injection compounds: Port A: 2.5–20 mM ADP (Sigma A2754); Port B: 40 µM Oligomycin (Sigma O4876); Port C: 2.5−320 µM BAM 15 (Tim Tec ST056388)/ 40−160 µM FCCP (Sigma C2920); Port D: 20 µM Antimycin A (Sigma A8674) and Rotenone (Sigma R8875).
**TIPS**: ADP can be prepared as 100 mM stock in MAS solution and adjusted to pH 7.2. Other injection compounds can be prepared at 1000 × in DMSO and diluted to 10 × with MAS for port loading. The final concentration in the well after injection is 1 × concentration.
• Complex I Assay Media: 11 mM Pyruvate (Sigma P2256), 11 mM malate (Sigma M1000), 11 mM L-proline (Sigma P0380), pH 7.2.
**TIPS**: Pyruvate, malate and L- proline can be prepared as 100 mM stock in MAS solution and adjusted to pH 7.2. The substrates can then be diluted to the desired concentration (11 mM) with MAS.

### Equipment

• pH meter
• Eppendorf centrifuge 5810R pre-cooled to 4°C
• Plate reader (SpectraMax M3)
• Non CO_2_ incubator set to 15°C
• XF24 Extracellular flux Analyzer (SeahorseBioscience, MA, USA) with the run temperature set as appropriate (we used 23°C).

## PROCEDURE

1. Mitochondrial isolation for Drosophila
  1.1. Collect 10 whole wandering third instar larvae and wash them in water and 70% ethanol (for this optimization we included males and females). Place them in 100 µl of ice-cold mitochondrial isolation buffer in 1.5 ml microcentrifuge tubes.
  1.2. Gently homogenize the third instar larvae or thoraces with 80 strokes up and down with a plastic microtube pestle.
**CAUTION**: Press dounce straight up and down without grinding. If a pellet forms during homogenization, release it with a spatula or pipette tip and continue the process.
  1.3. Transfer the homogenate into a 1-cc syringe containing 200 µl of isolation buffer.
  1.4. Force the homogenate through the cotton filter and collect in a new 1.5 ml microcentrifuge tube.
  1.5. Centrifuge the filtered homogenate at 1500 × g at 4°C for 8 min.
**CAUTION**: Centrifugation at a higher speed should be avoided as increasing the spin to 5000 × g results in mitochondria with low respiration rates.
  1.6. Remove and discard the cloudy supernatant, be careful not to disturb the pellet.
  1.7. Wash the pellet with 200 µl of mitochondrial isolation buffer and then resuspend in 20 µl of isolation buffer.
  1.8. Perform the Bradford assay according to the manufacturer’s instructions (Sigma B9616).
**TIPS**: We suggest 2 µl of mitochondrial stock be diluted with 10 µl of mitochondrial isolation buffer (1 to 6 dilutions) to ensure the readings are within the standard curve range for Bradford assay.
2. Prepare the XF sensor cartridge
  2.1. The day before the assay, hydrate the XF sensor cartridge at 23°C overnight in XF calibration buffer.
**NOTES**: The assay was performed at 23°C and temperature is subjective to change based on the study design.
  2.2. On the day of assay, load the XF sensor cartridge injections port with injections compounds.
  2.3. Volume of compounds loaded in each port: Port A: 50 µl; Port B: 55 µl; Port C: 60 µl; Port D: 65 µl.
  2.4. Calibrate the sensor cartridge by following the XF instrument protocol (**Table 1**).
3. Preparation of mitochondrial complex I assay plate
  3.1. Dilute the mitochondrial stock solution (obtained from step 1.7) with MAS to yield a final concentration of 0.02−0.4 µg/µl.
**TIPS**: Due to the high concentration of mitochondria in the suspension, it is recommended that the mitochondria be first mixed by gently pipetting, and then performing 1 to 6 dilutions with MAS to form a final volume of ~100µl (This additional step is subject to the mitochondrial stock concentration. E.g. a lower concentration of mitochondria may require 1 to 3 dilutions). This is then added to a larger volume to form the desired concentration.
  3.2. Pipette 50 µl of the diluted mitochondria into each well of XF cell culture microplate. *E.g.*, 50 µl of diluted mitochondria with 0.1, 0.2 and 0.4 µg/uL concentration will results in 5, 10 and 20 µg of total mitochondria per well.
  3.3. Transfer 50 µl of MAS into each background correction wells.
  3.4. Centrifuge the XF cell culture microplate at 2254 × g at 4°C for 20 min.
  3.5. After centrifugation, visualize the mitochondria under the microscope using 20 × magnification. Make note of any wells that do not have a “monolayer” of mitochondria adhered to the well bottom.
  3.6. Add 450 µl of complex I assay media to each well and incubate the cell culture microplate at 23°C for 10 min.
**NOTES**: The assay was performed at 23°C and temperature is subjective to change based on the study design.
  3.7. Subsequently, exchange the calibration plate with the cell culture microplate and follow the instructions on the instrument controller to continue with XF instrument protocol (**Table 1**).
**NOTES**: The waiting step in **Table 1** can be modified for a longer period if required.

## ANTICIPATED RESULTS

**Figure 1.**
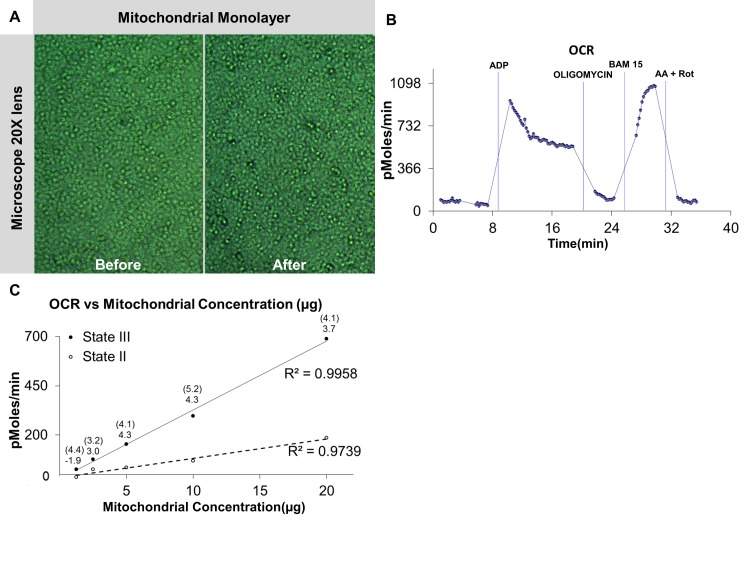
**Optimization of mitochondrial concentration for oxygen consumption rate (OCR). A.** Isolated mitochondria are visualized under the microscope before and after experiment. The mitochondria appear as a monolayer at the base of the well. **B.** Oxygen consumption rate in pmoles.min^−1^. The graph shown above is a typical plot obtained using the methods outlined in this protocol. The four injections are reported in blue. **C.** State II and III respiration rates are linear from 1.25 to 20 µg of mitochondrial protein. The number above each data point represents the RCR value calculated by state III/II and state III/IVo (value inside the bracket) for each mitochondrial concentration.

The typical microscopy image for the mitochondrial “monolayer” is shown in **Figure 1A**. A typical graph of oxygen consumption rate for Seahorse XF24 Extracellular Flux Analyzer is presented in **Figure 1B**. The results reveal that mitochondrial states II and III OCR are linear from 1.25 to 20 µg of mitochondrial protein (**Fig. 1C**). However, the optimum RCR values can be obtained using 5 and 10 µg of mitochondria. We recommend using 10 µg of mitochondria as the state II OCR falls within the range 50 pmoles.min^-1^< OCR < 200 pmoles.min^-1^. For the subsequent titration of the injection compounds, 10 µg of mitochondria was used in all wells.

**Figure 2.**
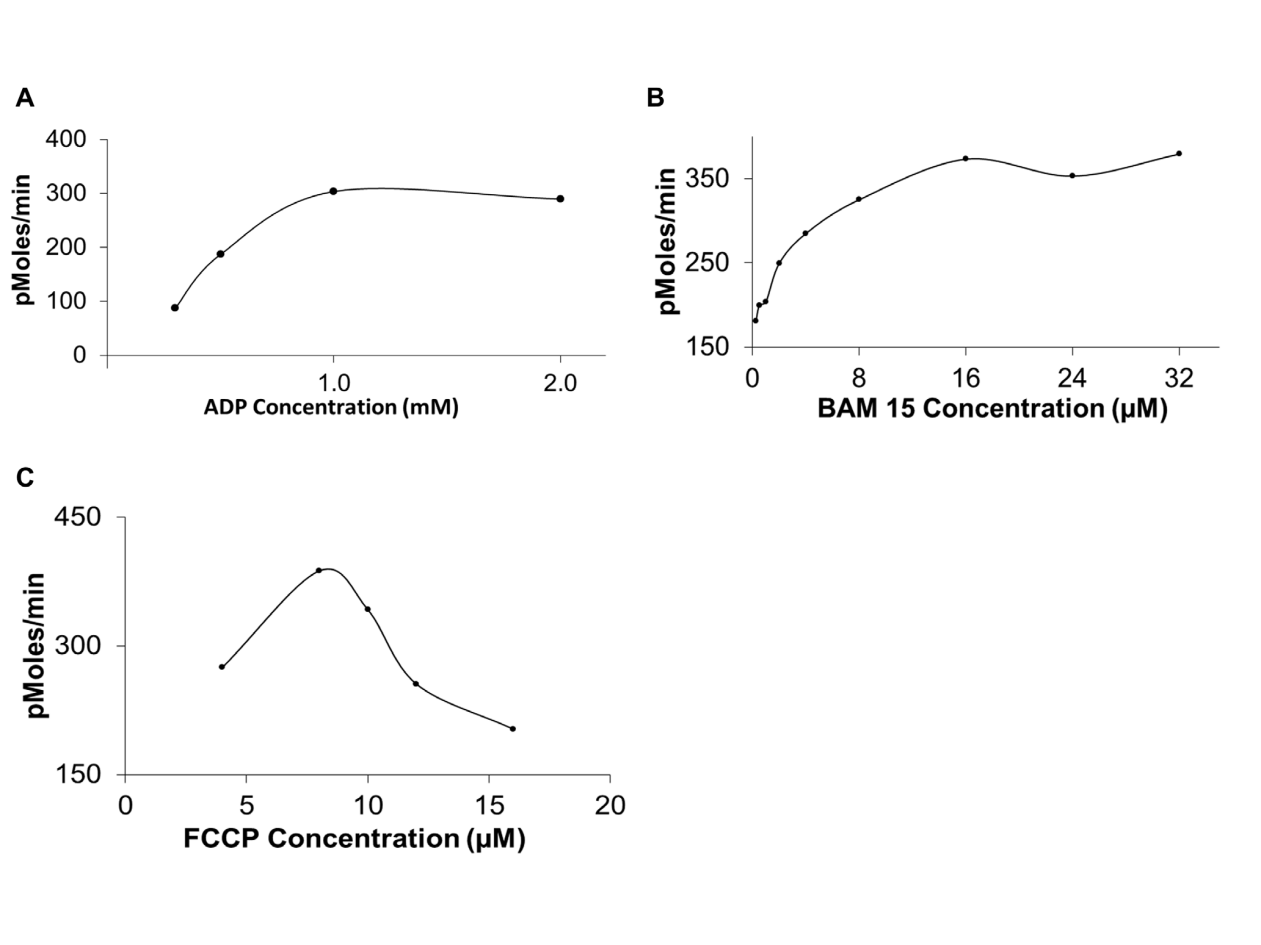
Optimization of injections. A. ADP is saturated at 1 mM concentration. B. BAM 15 is saturated at 16 µM and C. FCCP is saturated at 8 µM.

As shown in **Figure 2A**, the concentration of ADP is saturated at 1 mM concentration. ADP injection after 1 mM will not stimulate higher state III OCR. The optimum final well concentration for BAM 15 was 16 µM and for FCCP was 8 µM (**Fig. 2B** and C). Increasing the concentration of BAM 15 above the saturation point had no effects on maximum respiration. In contrast, an increase in FCCP above 8 µM reduces the maximum respiration. The results indicate that FCCP is cytotoxic to the mitochondria (above 8 µM) and higher concentrations depolarize the mitochondrial membrane [6].

This protocol was originally optimized for mitochondria extracted from third instar larvae. The optimized protocol for complex I mitochondrial respiration consists of 10 µg mitochondrial protein with the following injection for each port, Port A: 10 mM ADP; Port B: 40 µM oligomycin; Port C: 160 µM BAM 15 or 80 µM FCCP; Port D: 20 µM rotenone and antimycin A. Subsequently, we have been successful in applying this protocol to mitochondria extracted from adult thoraces after reducing the mitochondrial concentration to 5 µg.

Following initial preliminary studies, this final protocol was optimized in four steps (Mitochondrial concentration, ADP concentration, FCCP concentration and BAM15 concentration) in four separate plates. Where possible, we recommend researchers run each experimental treatment with at least four biological replicates each with three pseudo-replicates. Finally, to determine robustness of the method we replaced complex I assay media with 11 mM of succinate (Sigma S2378) and 11 mM of glycerol-3-phosphate (Sigma G7886). The basic protocol successfully determined complex II functions from mitochondria extracted from third instar larvae and complex III functions from mitochondria extracted from adult thoraces, respectively. Intriguingly, complex III functions from mitochondria extracted from third instar larvae and complex II functions from mitochondria extracted from adult thoraces failed suggesting a biological basis.

## TROUBLESHOOTING

Tips for troubleshooting can be found in **Table 2**.

**Table 2.** Troubleshooting.

Step	Problem	Possible reason	Solution
1.2	Isolated mitochondrial have a low RCR value.	• Mitochondrial membrane was disrupted during isolation. • Mitochondria pellet not washed thoroughly.	• Gently homogenize sample with straight up and down presses, without grinding. • Wash the mitochondria pellet twice.
1.8	Protein concentration was too high in Bradford assay.	• The sample was diluted with MAS.	• Dilute the sample with mitochondrial isolation buffer.
3.1	Low/high mitochondrial state II respiration	• Mitochondria concentration was too low/high.	• Adjusted concentration of mitochondria to 5-10 µg.
3.2	High variation of OCR between pseudo-replicate well.	• Mitochondria were not properly mixed before pipetting. • The mitochondria was not adhered to the well bottom.	• Mix the mitochondria solution by gently pipetting before loading into the well. • Check the mitochondria under microscope and exclude any wells that do not have a “monolayer” of mitochondria adhered to the well bottom.
